# Analysis of Staphylococcal Diversity in the Skin Microbiota of Healthy Riding Horses

**DOI:** 10.3390/antibiotics14101037

**Published:** 2025-10-16

**Authors:** Maria Wesołowska, Ewa Szczuka

**Affiliations:** Department of Microbiology, Institute of Experimental Biology, Faculty of Biology, Adam Mickiewicz University, Uniwersytetu Poznańskiego 6, 61-614 Poznań, Poland; maria.wesolowska@amu.edu.pl

**Keywords:** antimicrobial resistance, *Staphylococcus* species, equine skin microbiota, pathogens with zoonotic potential

## Abstract

**Background:** In animals, staphylococci constitute a significant part of the normal skin microbiota and mucous membranes. There is limited information available on staphylococci isolated from healthy horses. These skin-associated bacteria can be easily transferred between animals and horse riders via direct contact. Patients undergoing hippotherapy (i.e., medical or therapeutic sessions with horses) are especially at risk of being colonized by horse skin-associated bacteria. However, it remains unclear whether equine skin is colonized by antimicrobial-resistant (AMR) opportunistic pathogens, which may be of concern to human health. **Methods:** We cultivate staphylococci from samples collected from healthy, non-vet-visiting horses who live on private farms in a rural area. In total, 61 strains were isolated and identified at the species level using matrix-assisted laser desorption ionization time of flight mass spectrometry (MALDI-TOF MS). **Results:** The diversity of *Staphylococcus* species in the equine skin microbiota was relatively high and, with the exception of *S. aureus*, all the other recovered strains were coagulase-negative staphylococci (CoNS). In total, eleven different staphylococcal species were identified: *S. xylosus*, *S. sciuri*, *S. vitulinus*, *S. equorum*, *S. succinus*, *S. nepalensis*, *S. lentus*, *S. fleurettii*, *S. aureus*, *S. chromogenes*, and *S. simulans*. **Conclusions:** These results indicate that healthy equine skin is colonized by opportunistic pathogens that can be causative agents of infections that are also severe in humans. The resistance among the isolated strains was observed in eight antimicrobials of the total tested and 36% (22/61) of the isolates were resistant to at least one antimicrobial. However, their resistance to critically important antibiotics used in human medicine was low. Seven isolates (11.5%; 7/61) were classified as multidrug-resistant (MDR). *S. aureus* (1/61) showed MDR and was methicillin-resistant. The *S. aureus* isolate contained genes conferring resistance to antibiotics, i.e., β-lactams (*blaZ*, *mecA*), aminoglycosides (*aac*(*6*′)/*aph*(*2*″)), and macrolide–lincosamide–streptogramin B (*erm*(*B*), *erm*(*C*), and *lun*(*A*/*B*)). Also CoNS harbored genes conferring resistance to β-lactams (*blaZ*), aminoglycosides (*aac*(*6*′)/*aph*(*2*″), *ant*(*4*′)*-Ia*), MLSB (*erm*(*B*), *erm*(*C*), *lun*(*A*/*B*)), and tetracycline (*tetK*, *tetL*).

## 1. Introduction

Nowadays, horses are used for sports and recreational activities and play a significant role in therapy, especially for subjects affected by Down’ syndrome and autism spectrum disorder [1]. The close interaction between horses and riders, particularly during hippotherapy or care activities, may potentially facilitate the transmission of skin-associated bacteria [2]. Staphylococci are an important component of the skin and mucous membranes of animals [3]. There is limited information available on staphylococci isolated from healthy horses. Studies have focused on equine skin infection due to its relevance to animal health. In horses, *Staphylococcus aureus* has been implicated in various skin diseases and in more serious infections, such as pneumonia, bacteremia, osteomyelitis, and metritis [4,5]. *S. aureus* is also associated with EPD (equine pastern dermatitis), a multifunctional syndrome, manifesting as a skin lesion in the pastern areas [6]. It should be noted that *S. aureus* can be found in clinically healthy horses, but is not regarded as a typical commensal on the skin [7]. Other coagulase-positive staphylococci (CoPS), *S. intermedius* and *S. hyicus*, are reported as causative agents of skin and wound infections [8]. Coagulase-negative staphylococci (CoNS), e.g., *S. sciuri*, cause purulent skin lesions in horses [9]. Westgate et al. [10] reported *isolation* of different staphylococcal species: *S. aureus*, *S. auricularis*, *S. epidermidis*, *S. xylosus*, *S. sciuri*, *S. simulans*, *S. warneri*, *S. equorum*, *S. hominis*, and *S. pasteuri* from chronic equine wounds. Studies conducted by Kamus et al. [11] have shown that the equine skin microbiota is able to return to its initial composition after the wound healing process.

Methicillin-resistant staphylococci (MRS), particularly the methicillin-resistant *Staphylococcus aureus* (MRSA), pose a significant concern in veterinary medicine [12,13]. MRS are resistant to almost all beta-lactam antibiotics through the acquisition of methicillin-resistant genes (*mecA* or *mecC*) on the staphylococcal cassette chromosome *mec* (SCC*mec*). It is worth noting that MRS often exhibit multidrug resistance (i.e., to at least three non- beta-lactam antibiotics), which extremely limits therapeutic options [14].

As emphasized by the “One Health” approach, the microbiota of companion animals and humans influence each other. We think that patients undergoing hippotherapy are at risk of being colonized by horse skin-associated bacteria. Therefore, we conducted this research to answer the question whether multidrug-resistant opportunistic pathogens occur in a population of healthy horses. We also determined the staphylococcal species composition in the equine skin microbiota.

## 2. Results

### 2.1. Staphylococci Residing on the Skin and Constituting the Normal Equine Microbiota

A total of 61 staphylococcal isolates were obtained from 19 animals, with each individual yielding between two and five isolates. A wide variety of staphylococcal species (*n* = 11) was found, primarily *S. xylosus* (13/61, 21.3%), *S. sciuri* (13/61, 21.3%), *S. vitulinus* (12/61, 19.7%), and *S. equorum* (10/61, 16.5%), followed by *S. succinus* (7/61, 11,6%), *S. aureus* (1/61, 1.6%), *S. chromogenes* (1/61, 1.6%), *S. fleurettii* (1/61, 1.6%), *S. lentus* (1/61, 1.6%), *S. nepalensis* (1/61, 1.6%), and *S. simulans* (1/61, 1.6%) (Table 1).

### 2.2. Antibiotic Resistance

**Figure 1:**
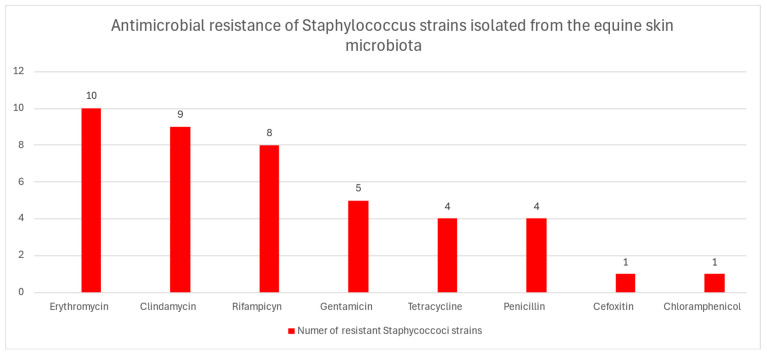
Antimicrobial resistance of *Staphylococcus* strains isolated from the equine skin microbiota.

Twenty two (36%) *Staphylococcus* strains were resistant to at least one antibiotic. The highest resistance rate was detected in the case of erythromycin (16.4%), clindamycin (14.7%), and rifampicin (13.1%). In addition, four strains were resistant to penicillin (6.6%) and tetracycline (6.6%) (Figure 1). A single isolate was resistant to cefoxitin (1.6%) and chloramphenicol (1.6%). All the *Staphylocoocus* strains were susceptible to levofloxacin, ciprofloxacin, tobramycin, amikacin, and tigecycline. The detailed results of antimicrobial resistance are presented in Table S1 and Table 2. Seven (11.5%) of these strains were resistant to at least three different classes of antimicrobials, and were thus considered multidrug-resistant. Importantly, the only strain of *S. aureus* isolated was methicillin-resistant. This *S. aureus* strain was resistant to cefoxitin, penicillin, gentamycin, erythromycin, clindamycin, and tetracycline and carried resistance determinants, i.e., *mecA*, *blaZ*, *aac*(*6*′)/*aph*(*2*″), *ermB*, *ermC*, and *lnu A*/*B* genes (Table 2). Genes coding for resistance to penicillin were also found in three strains of *S. succinus*, whereas genes coding for resistance to aminoglycoside were found in one *S. equorum* and two *S. sciuri* strains. Among genes conferring resistance to tetracycline, expected to be present in the tetracycline–resistant strains, only *tetL* and *tetK* were found in one *S. nepalensis* and in two *S. sciuri* isolates. Of note, the *ermB* (14/61) *and ermC* genes (11/61) were the most prevalent in the staphylococcal strains. The genes encoding resistance to macrolides (*ermB*, *ermC*) were also detected in six strains that expressed intermediate susceptibility or susceptibility to erythromycin. Three *Staphyloccous* strains with intermediate susceptibility to clindamycin, tested positive, using PCR, for the presence of *lnu*(*A*/*B*) gene. Similarly, two strains resistant to clindamycin carried the *lnu*(*A*/*B*) gene.

## 3. Discussion

In this study, we employed MALDI TOF to successfully characterize the population of staphylococci isolated from the equine skin microbiota. The horses included in this study had no history of hospitalization in a veterinary clinic or no clinical signs of infectious diseases and received no antibiotics in the past six months. In total, eleven different staphylococcal species were isolated: *S. xylosus*, *S. sciuri*, *S. vitulinus*, *S. equorum*, *S. succinus*, *S. nepalensis*, *S. lentus*, *S. fleurettii*, *S. aureus*, *S. chromogenes*, and *S. simulans*. To the best of our knowledge, this is the first study demonstrating such diversity in healthy horses. It should be noted that Madhaiyan et al. [15], based on the phylogenomic analyses of the *Staphylococcaceae* family, suggest the taxonomic reassignment of five *Staphylococcus* species, i.e., *Staphylococcus sciuri*, *Staphylococcus fleurettii*, *Staphylococcus lentus*, *Staphylococcus stepanovicii* and *Staphylococcus vitulinus*, to the novel genus *Mammaliicoccus* with *Mammaliicoccus sciuri* designated as the type species. Our study has revealed that the healthy equine skin is dominated by *S. xylosus*, *S. sciuri*, and *S. vitulinus*. Also, *S. equorum* and *S. succinus* are commonly found on the skin of the studied healthy individuals. A Japanese study also reported that *S. xylosus* and *S. sciuri* were isolated most frequently from the skin of horses [16]. However, *S. vitulinus*, *S. equorum*, and *S. succinus* were not detected on the equine skin. This diversity of the equine skin microbiota may be dependent on the geographical location of the animal. It should be noted that Matsuo et al. [16] used the16S-23S rDNA intergenic spacer PCR methods for the identification of staphylococci, which do not allow the identification of all species of staphylococci. Another study carried out in Switzerland, using phenotypic tests (API ID32STAPH), showed the colonization of the equine skin by *S. xylosus*, *S. vitulinus*, *S. equorum*, and *S. succinus*. However, these strains were isolated from the skin of healthy horses as well individuals hospitalized in an equine clinic [17]. In the present study, we also isolated *S. nepalensis*, *S. lentus*, *S. fleurettii*, *S. aureus*, *S. chromogenes*, and *S. simulans* strains from individual horses. With the exception of *S. aureus*, all the other recovered strains found are coagulase-negative staphylococci (CoNS). These findings seem to agree with previous reports confirming that most staphylococci isolated from the equine skin belonged to the CoNS [7,16,17]. One of the main limitations of this study is its focus on the staphylococcal composition of equine skin within a single geographical area. As a result, it is not possible to determine the influence of climate zones, environmental conditions, anthropogenic factors, diet, and other variables on the skin microbiota.

Close contact between animals and their owners can facilitate interspecies transmission. More recently, Uchida-Fujii et al. [18] reported the horse–veterinarian transmission of MRSA in an equine hospital. Although, 30% of the *human population are asymptomatically* colonized by *S. aureus*, this bacterium has the potential to cause a range of infections [19]. Of note, *S. aureus* is a leading cause of bacteremia, infective endocarditis, osteoarticular, skin and soft tissue, and device-related infections [19]. CoNS species are less virulent and are traditionally regarded as harmless skin commensals. However, in certain conditions such as the presence of foreign bodies and/or immunosuppression, CoNS may cause infections [20]. In particular, *S. sciuri* can cause serious infections in humans such as peritonitis, endocarditis, septic shock, urinary tract infections, and skin and soft tissue infections [21,22,23]. The other members of the *Staphylococcus sciuri* group, i.e., *S. vitulinus* and *S. lentus*, may cause urinary tract infections [23]. *S. lentus* has also been implicated in endocarditis, peritonitis, sinusitis, septic shock, and wound infections [24,25,26]. *S. xylosus* is classified as a nonpathogenic bacterium, but some strains can cause urinary tract infections and more rarely endocarditis, pyelonephritis, erythema nodosum, and orthopedic implant infections [27]. Recently, Brant et al. [28] reported a prosthetic knee joint infection in a 70-year old patient caused by *S. xylosus*. Also, *S. equorum*, and *S. suscinus* are rarely isolated from human clinical materials [29]. Interestingly, Khusro et al. [30] reported that the *S. suscinus* strain AAS2 shows anti-pathogenic activity against food-borne and enteric pathogens, i.e., *Staphylococcus aureus* and *Enterobacter aerogenes*. Special attention is given to *S. simulans* because this bacterium is seen as emerging cutaneous pathogen [31]. *S. simulans* has also been implicated in native valve endocarditis and neonatal sepsis [32,33]. Previously, Razonable et al. [34] reported a case of osteomyelitis and prosthetic joint infections due to *S. simulans* that developed in a farmer who had daily contact with cows. The information about *S. chromogenes* and *S. nepalensis* is sparse. However, there is a well-documented case of *S. nepalensis* bacteremia in a 71-year-old man [35]. In 2022, Yu et al. [33] reported that *S. chromogenes* was isolated from the blood of neonates with episodes of sepsis.

Since horses may be not only a source of staphylococcal infection but also a reservoir of bacteria carrying resistant and antibiotic genes, we have evaluated the bacterial susceptibility to antibiotics representing ten different classes. The isolates showed resistance to eight of the 15 tested antimicrobials, and 36% (22/61) of the isolates were resistant to at least one antibiotic. However, the resistance of the staphylococcal strains to critically important antibiotics used in human medicine (erythromycin, clindamycin, gentamycin, beta-lactams) remains very low or low. Karakulska et al. reported [36] that CoNS isolated from the nasal cavity of healthy horses were susceptible to most antimicrobial agents. Only, 17.2% of the isolates were resistant to one or two antimicrobial agents: β-lactams, erythromycin, gentamicin, and/or tetracycline. Studies conducted in Florida reported a statistically significant upward trend in penicillin resistance in *Staphylococcus* species over a 10-year period [4]. In our study, the staphylococcal strains showed a very low level (6.6%) of penicillin resistance. Unexpectedly, we identified one MRSA isolate. Studies from Canada and Brazil have reported no MRSA colonization in horses outside the hospital [37,38]. More recently, however, Kasper et al. [7] noted that MRSA were present among non-hospitalized horses in Germany. Although the resistance was not common among isolated staphylococci, we detected seven MDR isolates, i.e., five strains of *S. sciuri* and single strains of *S. aureus* and *S. equorum*. Significantly, higher prevalence (25%) of MDR has been reported among hospitalized horses in the UK [39]. In Australia, MDR was detected with a frequency of 12.8%. Importantly, only CoPS strains from equine clinical specimens were examined [40]. The present study also showed the presence of genes conferring resistance to antibiotics, i.e., β-lactams (*blaZ; mecA*), aminoglycosides (*aac*(*6*′)/*aph*(*2*″) *ant*(*4*′)*-Ia*), MLSB (*erm*(*B*), *erm*(*C*), and *lun*(*A*/*B*)), and tetracycline (*tetK*, *tetL*) among staphylococci colonizing healthy horses. However, the prevalence of these genes was low.

## 4. Methods

### 4.1. Sampling and Bacterial Identification

This study was carried out in strict accordance with the recommendations in the Guide for the National Ethical Committee for Experiments on Animals (KKE). Taking equine skin swabs does not require the consent of the KKE. Samples were collected from the skin of healthy, non-vet-visiting horses that did not receive any treatment (antibiotics). No skin lesions were observed in the horses, as confirmed by veterinarians. The horses lived on private farms in a rural area and were bred for sport/recreation activities and for therapy. In total, 19 horses were sampled. Skin cotton swabs (Sarstedt, Nümbrecht, Germany) moistened with sterile physiological saline (NaCl 0.85%) were placed in 2 mL of brain–heart infusion broth (BHI) (Oxoid, Basingstoke, UK) and incubated at 37 °C for 24 h. Then, 10 μL of this culture was inoculated on Mannitol Salt Agar (Biomeriux, France), and incubated in 37 °C for 24 h. Morphologically distinguishable staphylococcal colonies were picked with a needle, transferred to Columbia Agar (Biomeriux, France), and cultivated according to standard microbiological procedures. The preliminary identification of staphylococci was based on Gram staining and detection of catalase and coagulase production. Next, the bacteria were identified at the species level with MALDI-TOF MS (matrix-assisted laser desorption/ionization—time of flight mass spectrometry) following the manufacturer`s procedure (MALDI Biotyper^®^, Bruker, Billerica, MA, USA) [41].

### 4.2. Susceptibility Testing

The susceptibility of the strains was tested using antimicrobial disk diffusion tests (μg/disk) against 15 antimicrobials of 10 different classes: aminoglycosides (amikacin (30), gentamycin (10), tobramycin (10), beta-lactams (cefoxitin (30), penicillin G (10)), amphenicols (chloramphenicol (30)), fluoroquinolones (ciprofloxacin (5), levofloxacin (5), moxifloxacin (5)), macrolides (erythromycin (15), lincosamides (clindamycin (2), glycylcycline (tigecycline (15)), sulfonamides (trimethoprim/sulfamethoxazole (1.25 + 23.75), tetracyclines (tetracycline (30)), and rifamycins (rifampicin (5)). The CLSI recommendations were followed to perform the tests and interpretation of the results except for amikacin, tobramycin, and tigecycline. The CLSI guidelines do not provide interpretation criteria for these three antibiotics, and EUCAST recommendations were used in this case [42,43].

### 4.3. Preparation of Total DNA for PCR and Detection of Antimicrobial Resistance Genes

The total DNA was isolated and purified using the Genomic Mini DNA kit (A&A Biotechnology, Gdynia, Poland) according to the manufacturer’s instructions for Gram-positive bacteria. The presence of genes (*blaZ*, *mecA*, *tetK*, *tetM*, *tetL*, *tetO*, *aac*(*6*′)/*aph*(*2*″), *aph*(*30*)*-IIIa*, *ant*(*40*)*-Ia*, *erm*(*A*), *erm*(*B*), *erm*(*C*), *msr*(*A*), and *lun*(*A/B*) involved in resistance to various antibiotics was determined using the primers as previously described [44,45,46,47,48,49]. PCR amplification was carried out in a 50 µL reaction volume. The mixture consisted of 25 µL of 2× PCR Master Mix (Thermo Scientific™, Waltham, MA, USA), 20 pmol of each primer, and 50 ng of DNA template. The following type strains were used as a control: *S. aureus* NCTC 12,493 (positive control for *mecA*), *S. aureus* ATCC 29,213 (negative control for *mecA*, positive for *blaZ*), and *S. aureus* ATCC 25,923 (negative control for *blaZ*).

## 5. Conclusions

Our study provides detailed information on staphylococci residing on the skin that constitute normal equine microbiota. With the exception of *S. aureus*, all the other recovered strains were coagulase-negative staphylococci (CoNS), i.e., *S. sciuri*, *S. xylosus*, *S. vitulinus*, *S. equorum*, *S. succinus*, *S. nepalensis*, *S. lentus*, *S. fleurettii*, *S. chromogenes*, and *S. simulans*. These CoNS species are regarded as harmless skin commensals. However, in certain conditions such as the presence of foreign bodies and/or immunosuppression, CoNS may cause infections. Notably, only seven strains (11.5%) exhibited multidrug resistance (MDR).

## Figures and Tables

**Table 1:** *Stahylococcus* species isolated from the equine skin microbiota.

Identified *Stapylococcus* Species	Number of Strains	%
*S. aureus*	1	1.64
*S. chromogenes*	1	1.64
*S. equorum*	10	16.39
*S. fleurettii*	1	1.64
*S. lentus*	1	1.64
*S. nepalensis*	1	1.64
*S. sciuri*	13	21.31
*S. simulans*	1	1.64
*S. succinus*	7	11.48
*S. vitulinus*	12	19.67
*S. xylosus*	13	21.31
Overall	61	100.00

**Table 2:** Antimicrobial resistance profiles and distribution of resistance genes in isolated staphylococcal isolates.

**Strain**	**Isolate ID**	Antimicrobial Resistance Profile	*mecA*	*blaZ*	*tetK*	*tetM*	*tetL*	*tetO*	*aac*(*6*′)/ *aph*(*2*″)	*aph*(*30*)*-IIIa*	*ant*(*40*)*-Ia*	*ermA*	*ermB*	*ermC*	*msr*(*A*)	*lnu*(*A*/*B*)
*S. vitulinus*	H11	RA	0	0	0	0	0	0	0	0	0	0	0	0	0	0
*S. xylosus*	H18	RA	0	0	0	0	0	0	0	0	0	0	0	0	0	0
*S. sciuri*	H28	Tet	0	0	1	0	0	0	0	0	0	0	0	0	0	0
*S. sciuri*	H.29	CC	0	0	0	0	0	0	0	0	0	0	1	0	0	1
*S. sciuri*	H.30	GN, E, RA	0	0	0	0	0	0	0	0	0	0	1	1	0	0
*S. sciuri*	H.31	GN, E. CC, RA	0	0	0	0	0	0	1	0	0	0	1	1	0	0
*S. sciuri*	H.32	GN, E. CC, RA	0	0	0	0	0	0	1	0	0	0	1	1	0	0
*S. sciuri*	H.34	E, CC	0	0	0	0	0	0	0	0	1	0	1	1	0	0
*S. sciuri*	H36	CC, Tet, P	0	0	0	0	1	0	0	0	1	0	0	0	0	0
*S. sciuri*	H37	E, CC, P	0	0	0	0	0	0	0	0	0	0	1	1	0	0
*S. equorum*	H39	E	0	0	0	0	0	0	0	0	0	0	1	1	0	0
*S. equorum*	H40	E, CC	0	0	0	0	0	0	0	0	0	0	0	1	0	0
*S. equorum*	H42	GN	0	0	0	0	0	0	0	0	1	0	0	0	0	0
*S. equorum*	H44	E, RA	0	0	0	0	0	0	0	0	0	0	0	0	0	0
*S. equorum*	H45	E, RA, C	0	0	0	0	0	0	0	0	0	0	1	1	0	0
*S. succinus*	H52	P	0	1	0	0	0	0	0	0	0	0	0	0	0	0
*S. succinus*	H53	P	0	1	0	0	0	0	0	0	0	0	0	0	0	0
*S. succinus*	H54	P	0	1	0	0	0	0	0	0	0	0	0	0	0	0
*S. nepalensis*	H56	Tet	0	0	1	0	0	0	0	0	0	0	0	0	0	0
*S. lentus*	H57	CC	0	0	0	0	0	0	0	0	0	0	0	0	0	1
*S. aureus*	H59	FOX E, CC, Tet, GN, P	1	1	0	0	0	0	1	0	0	0	1	1	0	1
*S. chromogenes*	H60	RA	0	0	0	0	0	0	0	0	0	0	0	0	0	0

Only antibiotic-resistant strains are included in the table. 1—presence of resistance gene, 0—lack of resistance gene, P—penicillin, E—erythromycin, Tet—tetracycline, CC—clindamycin, GN—gentamycin, RA—rifampicin, C—chloramphenicol.

## Data Availability

The original contributions presented in this study are included in the article. Further inquiries can be directed to the corresponding author.

## Supplementary Materials

The following supporting information can be downloaded at: https://www.mdpi.com/article/10.3390/antibiotics14101037/s1, Table S1: Antimicrobial resistance of *Staphylococcus* strains isolated from the equine skin microbiota.

## References

[1] Lönker N.S., Fechner K., Wahed A.A.E. (2020). Horses as a crucial part of One Health. Vet. Sci..

[2] Khairullah A.R., Sudjarwo S.A., Effendi M.H., Ramandinianto S.C., Widodo A., Riwu K.H.P. (2022). A review of horses as a source of spreading livestock-associated methicillin-resistant *Staphylococcus aureus* to human health. Vet. World.

[3] Cosseau C., Romano-Bertrand S., Duplan H., Lucas O., Ingrassia I., Pigasse C., Roques C., Jumas-Bilakb E. (2016). Proteobacteria from the human skin microbiota: Species-level diversity and hypotheses. One Health.

[4] Marshall K., Marsella R. (2023). Evolution of the prevalence of antibiotic resistance to *Staphylococcus* spp. isolated from horses in Florida over a 10-year period. Vet. Sci..

[5] Sauvé F. (2021). Staphylococcal cutaneous infection in horses: From the early 2000s to the present. Can. Vet. J..

[6] Kaiser-Thom S., Gerber V., Collaud A., Hurni J., Perreten V. (2022). Prevalence and WGS-based characteristics of *Staphylococcus aureus* in the nasal mucosa and pastern of horses with equine pastern dermatitis. BMC Vet. Res..

[7] Kaspar U., Lützau K., Schlattmann A., Rösler U., Köck R., Becker K. (2019). Zoonotic multidrug-resistant microorganisms among non-hospitalized horses from Germany. One Health.

[8] White S.D. (2005). Equine bacterial and fungal diseases: A diagnostic and therapeutic update. Clin. Tech. Equine Pract..

[9] Beims H., Overmann A., Fulde M., Steinert M., Bergmann S. (2016). Isolation of *Staphylococcus sciuri* from horse skin infection. Open Vet. J..

[10] Westgate S.J., Percival S.L., Knottenbelt D.C., Clegg P.D., Cochrane C.A. (2011). Microbiology of equine wounds and evidence of bacterial biofilms. Vet. Microbiol..

[11] Kamus L.J., Theoret C., Costa M.C. (2018). Use of next generation sequencing to investigate the microbiota of experimentally induced wounds and the effect of bandaging in horses. PLoS ONE.

[12] Schwarz S., Feßler A.T., Loncaric I., Wu C., Kadlec K., Wang Y., Shen J. (2018). Antimicrobial resistance among staphylococci of animal origin. Microbiol. Spectr..

[13] Isgren C.M. (2022). Improving clinical outcomes via responsible antimicrobial use in horses. Equine Vet. Educ..

[14] Aires-de-Sousa M. (2017). Methicillin-resistant *Staphylococcus aureus* among animals: Current overview. Clin. Microbiol. Infect..

[15] Madhaiyan M., Wirth J.S., Saravanan V.S. (2020). Phylogenomic analyses of the *Staphylococcaceae* family suggest the reclassification of five species within the genus *Staphylococcus* as heterotypic synonyms, the promotion of five subspecies to novel species, the taxonomic reassignment of five *Staphylococcus* species to *Mammaliicoccus* gen. nov., and the formal assignment of *Nosocomiicoccus* to the family *Staphylococcaceae*. Int. J. Syst. Evol. Microbiol..

[16] Matsuo E., Kawano J., Yasuda R., Takagi M., Shimizu A., Anzai T., Hashikura S. (2001). Species distribution of Staphylococci in the nares and skin of horses. J. Equine Sci..

[17] Schnellmann C., Gerber V., Rossano A., Jaquier V., Panchaud Y., Doherr M.G., Thomann A., Straub R., Perreten V. (2006). Presence of new *mecA* and *mph(C)* variants conferring antibiotic resistance in *Staphylococcus* spp. isolated from the skin of horses before and after clinic admission. J. Clin. Microbiol..

[18] Uchida-Fujii E., Niwa H., Kanai K., Kinoshita Y., Kuroda T., Toshio Nukada T., Takanori Ueno T. (2022). Outbreak of methicillin-resistant *Staphylococcus aureus* sequence type 1, *spa* type t1784, in an equine hospital in Japan. Vet. Anim. Sci..

[19] Ahmad-Mansour N., Loubet P., Pouget C., Dunyach-Remy C., Sotto A., Lavigne J.P., Molle V. (2021). *Staphylococcus aureus* toxins: An update on their pathogenic properties and potential treatments. Toxins.

[20] Swaney M.H., Kalan L.R. (2021). Living in your skin: Microbes, molecules, and mechanisms. Infect. Immun..

[21] Shittu A., Lin J., Morrison D., Kolawole D. (2004). Isolation and molecular characterization of multiresistant *Staphylococcus sciuri* and *Staphylococcus haemolyticus* associated with skin and soft-tissue infections. J. Med. Microbiol..

[22] Stepanovic S., Dakic I., Morrison D., Hauschild T., Jezek P., Petrás P., Martel A., Vukovic D., Shittu A., Devriese L.A. (2005). Identification and characterization of clinical isolates of members of the *Staphylococcus sciuri* group. J. Clin. Microbiol..

[23] Stepanovic S., Jezek P., Vukovic D., Dakic I., Petras P. (2003). Isolation of members of the *Staphylococcus sciuri* group from urine and their relationship to urinary tract infections. J. Clin. Microbiol..

[24] Mazal C., Sieger B. (2010). *Staphylococcus lentus*: The troublemaker. Int. J. Infect. Dis..

[25] Rivera M., Dominguez M.D., Mendiola N.R., Roso G.R., Quereda C. (2014). *Staphylococcus lentus* peritonitis: A case report. Perit. Dial. Int..

[26] Hay C.Y., Sherris D.A. (2020). *Staphylococcus lentus* sinusitis: A new sinonasal pathogen. Ear Nose Throat J..

[27] Giordano N., Corallo C., Miracco C., Papakostas P., Montella A., Figura N., Nuti R. (2016). Erythema nodosum associated with *Staphylococcus xylosus* septicemia. J. Microbiol. Immunol. Infect..

[28] Brand Y.E., Rufer B. (2021). Late prosthetic knee joint infection with *Staphylococcus xylosus*. ID Cases.

[29] Novaková D., Sedláček I., Pantuček R., Štètina V., Švec P., Petráš P. (2006). *Staphylococcus equorum* and *Staphylococcus succinus* isolated from human clinical specimens. J. Med. Microbiol..

[30] Khusro A., Aarti C., Barbabosa-Pilego A., Hernández S.H. (2019). Anti-pathogenic, antibiofilm, and technological properties of fermented food associated *Staphylococcus succinus* strain AAS2. Prep. Biochem. Biotechnol..

[31] Shields B.E., Tschetter A.J., Wanat K.A. (2016). *Staphylococcus simulans*: An emerging cutaneous pathogen. JAAD Case Rep..

[32] Vallianou N., Evangelopoulos A., Makri P., Zacharias G., Stefanitsi P., Karachalios A. (2008). Vertebral osteomyelitis and native valve endocarditis due to *Staphylococcus simulans*: A case report. J. Med. Case Rep..

[33] Yu Y., Dong Q., Li S., Qi H., Tan X. (2022). Etiology and clinical characteristics of neonatal sepsis in different medical setting models: A retrospective multi-center study. Front. Pediatr..

[34] Razonable R.R., Lewallen D.G., Patel R., Osmon D.R. (2001). Vertebral osteomyelitis and prosthetic joint infection due to *Staphylococcus simulans*. Mayo. Clin. Proc..

[35] Hosoya S., Kutsuna S., Shiojiri D., Tamura S., Isaka E., Wakimoto Y. (2020). *Leuconostoc lactis* and *Staphylococcus nepalensis* bacteremia, Japan. Emerg. Infect. Dis..

[36] Karakulska J., Fijałkowski K., Nawrotek P., Pobucewicz A., Poszumski F., Czernomysy-Furowicz D. (2012). Identification and methicillin resistance of coagulase-negative staphylococci isolated from nasal cavity of healthy horses. J. Microbiol..

[37] Burton S., Reid-Smith R., McClure J.T., Weese J.S. (2008). *Staphylococcus aureus* colonization in healthy horses in Atlantic Canada. Can. Vet. J..

[38] Mota S.L., dos Santos L.O., Vidaletti M.R., Rodrigues R.O., Coppola M.M., Mayer F.Q. (2021). Antimicrobial resistance of coagulase-positive Staphylococcus isolated from healthy Crioulo horses and associated risk factors. J. Equine Vet. Sci..

[39] Isgren C.M., Williams N.J., Fletcher O.D., Timofte D., Newton R.J., Maddox T.W. (2021). Antimicrobial resistance in clinical bacterial isolates from horses in the UK. Equine Vet. J..

[40] Saputra S., Jordan D., Worthing K.A., Norris J.M., Wong H.S., Abraham R. (2017). Antimicrobial resistance in coagulase-positive staphylococci isolated from companion animals in Australia: A one year study. PLoS ONE.

[41] Dubois D., Leyssene D., Chacornac J.P., Kostrzewa M., Schmit P.O., Talon R., Bonnet R., Delmas J. (2009). Identification of a variety of *Staphylococcus* species by Matrix-Assisted Laser Desorption Ionization-Time of Flight Mass Spectrometry. J. Clin. Microbiol..

[42] CLSI M100-ED32:2022; Performance Standards for Antimicrobial Susceptibility Testing, 34th Ed. https://clsi.org/shop/standards/m100/.

[43] European Committee on Antimicrobial Susceptibility Testing (EUCAST) (2025). Breakpoint Tables for Interpretation of MICs and Zone Diameters, Version 15.0. https://www.eucast.org.

[44] Sawant A.A., Gillespie B.E., Oliver S.P. (2009). Antimicrobial susceptibility of coagulase-negative *Staphylococcus* species isolated from bovine milk. Vet. Microbiol..

[45] Nawaz M., Khan S.A., Khan A.A., Khambaty F.M., Cerniglia C.E. (2000). Comparative molecular analysis of erythromycin-resistance determinants in staphylococcal isolates of poultry and human origin. Mol. Cell. Probes.

[46] Chajęcka-Wierzchowska W., Zadernowska A., Nalepa B., Sierpińska M., Łaniewska-Trokenheim L. (2015). Coagulase-negative staphylococci (CoNS) isolated from ready-to-eat food of animal origin--phenotypic and genotypic antibiotic resistance. Food Microbiol..

[47] Gómez-Sanz E., Torres C., Lozano C., Fernández-Pérez R., Aspiroz C., Ruiz-Larrea F., Zarazaga M. (2010). Detection, molecular characterization, and clonal diversity of methicillin-resistant *Staphylococcus aureus* CC398 and CC97 in Spanish slaughter pigs of different age groups. Foodborne Pathog. Dis..

[48] Ardic N., Sareyyupoglu B., Ozyurt M., Haznedaroglu T., Ilga U. (2006). Investigation of aminoglycoside modifying enzyme genes in methicillin-resistant staphylococci. Microbiol. Res..

[49] Le Bouter A., Leclercq R., Cattoir V. (2011). Molecular basis of resistance to macrolides, lincosamides and streptogramins in *Staphylococcus saprophyticus* clinical isolates. Int. J. Antimicrob. Agents.

