# Supplementary material for: Analysis of Staphylococcal Diversity in the Skin Microbiota of Healthy Riding Horses
Source: Antibiotics (Basel). 2025 Oct 16;14(10):1037. doi: 10.3390/antibiotics14101037 (PMC12561800; doi:10.3390/antibiotics14101037)
Supplement: Supplementary file 1 [file antibiotics-14-01037-s001.zip › antibiotics-3883449-supplementary.pdf]

Table S1. Antimicrobial resistance of *Staphylococcus* strains isolated from the equine skin microbiota.

**Antibiotic Class**	**Antimicrobial Drug**	**Antimicrobial Susceptibility**					
		**Resistant**		**Intermediate**		**Sensitive**	
		n	**%**	***n***	**%**	***n***	**%**
β-lactams	Cefoxitin	1	1.64	0	00.00	60	98.36
	Penicillin	4	6.56	0	00.00	56	91.80
Macrolides	Erythromycin	10	16.39	6	9.83	45	73.77
Lincosamides	Clindamycin	9	14.75	12	19.67	40	65.57
Tetracyklines	Tetracycline	4	6.56	1	1.64	56	91.80
Aminoglycosides	Amikacin	0	00.00	0	00.00	61	100.00
	Gentamycin	5	8.19	0	00.00	56	91.80
	Tobramycin	0	00.00	0	00.00	61	100.00
Fluoroquinolones	Ciprofloxacin	0	00.00	0	00.00	61	100.00
	Levofloxacin	0	00.00	0	00.00	61	100.00
	Moxifloxacin	0	00.00	2	3.28	59	96.72
Ryfamicins	Rifampicin	8	13.10	0	00.00	53	86.88
Chloramphenicol	Chloramphenicol	1	1.64	0	00.00	60	98.36
Sulphonamides	Trimetoprim–sulfametoksaole	0	00.00	1	1.64	60	98.36
Glycylcyclines	Tigecycline	0	00.00	0	00.00	61	100.00
